# Schwann cell-like differentiated adipose stem cells promote neurite outgrowth via secreted exosomes and RNA transfer

**DOI:** 10.1186/s13287-018-1017-8

**Published:** 2018-10-11

**Authors:** Rosanna C Ching, Mikael Wiberg, Paul J Kingham

**Affiliations:** 1 Department of Integrative Medical Biology, Section for Anatomy, Umeå University, 901 87 Umeå, Sweden; 2 Department of Surgical and Perioperative Sciences, Hand and Plastic Surgery, Umeå University, Umeå, Sweden

**Keywords:** Exosomes, Extracellular vesicles, Peripheral nerves, Regeneration, Schwann cells, Stem cells

## Abstract

**Background:**

Adipose derived stem cells can be stimulated to produce a growth factor rich secretome which enhances axon regeneration. In this study we investigated the importance of exosomes, extracellular vesicles released by many different cell types, including stem cells and endogenous nervous system Schwann cells (SCs), on neurite outgrowth.

**Methods:**

Adipose derived stem cells were differentiated towards a Schwann cell-like phenotype (dADSCs) by *in vitro* stimulation with a mix of factors (basic fibroblast growth factor, platelet derived growth factor-AA, neuregulin-1 and forskolin). Using a precipitation and low-speed centrifugation protocol the extracellular vesicles were isolated from the medium of the stem cells cultures and also from primary SCs. The conditioned media or concentrated vesicles were applied to neurons *in vitro* and computerised image analysis was used to assess neurite outgrowth. Total RNA was purified from the extracellular vesicles and investigated using qRT-PCR.

**Results:**

Application of exosomes derived from SCs significantly enhanced *in vitro* neurite outgrowth and this was replicated by the exosomes from dADSCs. qRT-PCR demonstrated that the exosomes contained mRNAs and miRNAs known to play a role in nerve regeneration and these molecules were up-regulated by the Schwann cell differentiation protocol. Transfer of fluorescently tagged exosomal RNA to neurons was detected and destruction of the RNA by UV-irradiation significantly reduced the dADSCs exosome effects on neurite outgrowth. In contrast, this process had no significant effect on the SCs-derived exosomes.

**Conclusions:**

In summary, this work suggests that stem cell-derived exosomes might be a useful adjunct to other novel therapeutic interventions in nerve repair.

## Background

Peripheral nerve injury evokes a significant molecular and cellular response involving both the damaged neurons and supporting Schwann cells (SCs). Distal to the lesion, axons degenerate and the SCs dedifferentiate into a non-myelinating repair phenotype [1] which then proliferate and form the bands of Büngner to help guide the regenerating proximal axons. This process is slow, occurring over approximately two weeks [2] and the subsequent axon re-growth is limited to approximately 1 mm/day [3]. Patients with nerve gap defects have limited recovery, as regenerating axons must traverse the gap without any structural support and with only limited input from the SCs. The current gold standard treatment involves harvesting a healthy functioning nerve from elsewhere on the patient and placing it as a graft at the site of the nerve gap. Other options involve using synthetic nerve guidance conduits, but their lack of biological and cellular support means sacrificing a functioning nerve is still considered superior. One way to overcome these limitations is to impregnate the conduits with SCs or stem cells, both of which have shown ability to boost axon regeneration [4–6]. This improvement is, in large part, coordinated by the secretion of pro-regenerative growth factors and cytokines. An ideal scenario to treat patients with minimal delay, would be to have an “off-the shelf” supply of the secretome, which could be combined with the nerve guidance conduits to promote rapid axon regeneration.

In addition to conventional secreted paracrine molecules with short half-life e.g. neurotrophic factors, the cell secretome contains exosomes; extracellular vesicles with a diameter size of 10–150 nm [7–10] constructed of a phospholipid bilayer membrane which wraps and protects their cargo of RNAs, proteins and lipids. These immunologically inert [11] nanoparticles transport the cargo from a parent cell to targeted recipient cells where they are internalised and their contents processed. Interestingly, the RNA that is transferred has been shown to affect protein production at the recipient cell and as such signifies a newly identified method of horizontal gene transfer [12, 13]. Exosomes represent one kind of extracellular vesicle, which is a heterogeneous population including larger vesicles such as microvesicles [14]. Each subclass are formed by different cellular pathways but still transfer substances to distant cells, and it has not yet been possible to definitively separate the various vesicles from each other [15]. This, coupled with different understandings of the terminology used in the literature, has led to the call for explicit descriptors when describing the vesicles [16]. In this study we use the term exosome to refer to all the extracellular vesicles isolated using our described methods and found to be within the size range described above.

SCs have recently been found to secrete exosomes [17] which enhance axonal regeneration both *in vitro* and *in vivo* [18]. The SC exosomes are selectively internalised by peripheral nerve axons [18] and as such indicate a likely specificity of their cargo in the development, protection or regeneration of the peripheral nervous system. However, the cargo and its effect on neurons have yet to be explored. Our previous work has shown how adipose-derived stem cells (ADSCs) can be differentiated towards a Schwann-cell like phenotype (dADSCs) [19], and as such it is possible that these cells produce similar exosomes to SCs, with similar cargo that may also promote axonal re-growth. Thus, the aim of this study was to compare dADSC and SC-derived exosomes and examine their effects on neuronal outgrowth.

## Methods

### Cell harvest and culture

Adipose derived stem cells were isolated from adult Sprague Dawley rats as previously described [19]. The animal care and experimental procedures were carried out in accordance with the Directive 2010/63/EU of the European Parliament and of the Council on the protection of animals used for scientific purposes and was also approved by the Northern Swedish Committee for Ethics in Animal Experiments (No. A186–12). In brief, the stromal vascular fraction pellet obtained after tissue enzyme digestion and centrifugation was plated in growth medium containing Minimal Essential Medium-alpha (MEM-α; Invitrogen) with 10% foetal calf serum (FCS; Sigma-Aldrich) and 1% penicillin-streptomycin (PAA). Cultures were maintained at 37 °C and 5% CO_2_. For the first 3 days of culture the cells were washed daily with Hanks Balanced Salt Solution to remove all non-adherent cells. At passage two the cells were differentiated into a Schwann-cell-like phenotype (dADSCs) in two initial steps, firstly by replacing the growth medium with medium supplemented with 1 mM β-mercaptoethanol (Scharlau Chemicals) for 24 h and then by treating the cells with 35 ng/ml all-trans-retinoic acid (Sigma-Aldrich) for 72 h. Thereafter the cells were treated with differentiating medium consisting of growth medium supplemented with 5 ng/ml platelet-derived growth factor (PeproTech), 10 ng/ml basic fibroblast growth factor (PeproTech), 14 μM forskolin (Sigma-Aldrich) and 252 ng/ml neuregulin-1 (R&D Systems) for a minimum of 14 days before characterisation (see next section). The added growth factors were selected on the basis of their roles in modulating Schwann cell development and survival and the above described protocol was based on a model first described by Dezawa *et al.* for the differentiation of bone marrow derived stem/stromal cells [20]. Primary Schwann cells (SCs) were isolated from rat sciatic nerves and cultured in Dulbecco’s Modified Eagle’s Medium (DMEM; Invitrogen) containing 10% (*v*/v) FCS, 1% (*v*/v) penicillin/streptomycin, 14 μM forskolin and 100 ng/ml neuregulin-1 as previously described [21]. The NG108–15 cell line (ATCC) was used for neurite outgrowth assays [19]. The cells were cultured in DMEM with 10% (v/v) FCS and 1% (v/v) penicillin/streptomycin.

### Stem cell characterisation

Immunostaining was performed on undifferentiated stem cells (uADSCs) at passage 2 cultured on LabTek™ (Nunc) slides. After blocking with normal serum, the primary antibodies were applied for 2 h at room temperature CD73 (1:100; BD Biosciences), CD90 (1:1000; Millipore), CD105 (1:20; Abcam Ltd) and CD34 (1:50; Santa Cruz Biotechnology). After rinsing in phosphate-buffered saline, either secondary goat anti-mouse or donkey anti-goat Alexa Fluor 488 conjugated antibodies (1:300; ThermoFisher) were applied for 1 h at room temperature in the dark. The slides were then cover-slipped with ProLong mounting media containing 4–6-diamido-2-phenylindole (DAPI; ThermoFisher). The specificity of staining was tested by omission of the primary antibodies. To confirm multi-potency the uADSCs were treated with either adipogenic or osteogenic supplements according to the protocol described by the manufacturer (Rat Mesenchymal Stem Cell Functional Identification Kit, R & D Systems). Stem cells which were induced to a Schwann cell-like phenotype were immunostained with Sox-10 (1:200; R&D Systems), S100 protein (1:2000; Dako) and glial fibrillary acidic protein (GFAP 1:1000; Dako) antibodies. For comparison, uADSCs and primary Schwann cells were stained under identical conditions.

### Exosome isolation and characterisation

SCs, uADSCs and dADSCs were each cultured at 4 × 10^6^ cells/75cm^3^ density in medium containing exosome-free FCS (Sanbio, Netherlands) for 48–72 h prior to harvesting the resultant conditioned media from the cultures. Some of the conditioned medium was first tested for biological activity by application to NG108–15 neurons (see next section). Next a precipitation method of exosome isolation was chosen due to the ease and speed of the technique as well as the high yield of exosomes it produces [22]. Thus, a commercially available kit was used according to the manufacturer’s protocol (Total Exosome Isolation Reagent; Invitrogen). The resultant exosome pellet was resuspended in either 100 μl of phosphate buffer saline (PBS; used for exosome characterisation), DMEM (used in neurite outgrowth assays) or Invitrogen exosome resuspension buffer (used for RNA extraction). Nanoparticle tracking analyses (Malvern Instruments) was used to confirm the size of the isolated extracellular vesicles. For Transmission Electron Microscopy (TEM) aliquots from exosome preparations were deposited onto formvar and carbon coated 300 mesh copper grids for 1.5 min at room temperature and thereafter stained with 1.5% uranyl acetate (3 × 10 s with blotting). The grids were imaged using a JEM-1400 (Jeol Ltd.), 120KV electron microscope. Western blotting was also used to detect recognised exosomal markers. In brief, exosomes were lysed in RIPA buffer and total protein was quantified using the BioRad Dc Protein Assay (Bio-Rad Laboratories). Samples were run on 10% (*v*/v) polyacrylamide gels and then the proteins were transferred to nitrocellulose membranes for 60 min at 80 V. The membranes were probed with CD63 antibody (Santa Cruz Biotechnology) and HSP70 antibody (Santa Cruz Biotechnology).

### Neurite outgrowth experiments

NG108–15 neurons were seeded at a density of 1000cells/2cm^2^ and allowed to adhere to the tissue culture plastic for at least 6 h prior to the culture media being changed according to various experimental conditions. In a first series of experiments, cell conditioned media was collected after 48 h from SCs, uADSCs and dADSCs (4 × 10^6^ cells/75cm^2^ flask). An additional group was made, whereby the dADSCs were cultured for 72 h in medium devoid of their stimulating factors (de-dADSCs). Control media (no additional growth factors), or control SCs or dADSCs media (with relevant stimulating factors), which had not been exposed to the cells but had been prepared and incubated for the same duration, were also collected. The conditioned media and controls were applied directly to the NG108–15 cells for 24 h. Each treatment was performed in triplicate and the conditioned media used was from three independent rat cell cultures (with matching rats for each SCs and stem cells preparation). After 24 h in culture, photographs were taken either via light microscopy, or after fixation using 4% (*v*/v) paraformaldehyde the cultures were immunofluorescently labelled with βIII-tubulin antibody [6]. A minimum of four areas with clearly defined isolated neurons per well were traced using Image ProPlus software (Media Cybernetics) to measure the longest neurites. In the next series of experiments, we sought to determine the role of exosomes found in the conditioned media. Exosomes isolated from uADSCs, dADSCs or SCs were resuspended in 100 μl DMEM. The experimental media applied to the NG108–15 neurons was made up of 100 μl exosomes in DMEM and 800 μl standard NG108–15 media; the resultant 900 μl mixture for each animal and cell-type was then divided across three replicates. An additional control to those mentioned above was used, whereby 100 μl of DMEM not containing exosomes was applied to the cells. Cultures were maintained for 24 h before analysis as described above. These experiments were performed 3–4 times. A dose response of exosomes, according to their protein content, indicated that a minimum threshold of 100-150 μg was necessary to elicit significant increases in neurite outgrowth. To test if the effects of exosomes on neurite outgrowth could be mediated by RNA transfer, in some experiments we also first exposed exosomes to UV-light for 2 × 30 min, as UV-light inactivates exosomal RNA functions [23, 24] and then added the exosomes to the NG108–15 cells as above. In a further experiment, exosome proteins were denatured by heating to 98 °C for 10 min, allowed to cool and then added to the NG108–15 cells.

### Exosomal RNA extraction and identification

RNA (mRNAs and miRNAs) were isolated from the exosomes using the Total Exosome RNA and Protein Isolation Kit (Invitrogen) according to the manufacturer’s instructions. The quantity of RNA in 100 μl of elution solution was measured using a NanoDrop device (ThermoFisher) and then 10 ng of total RNA per reaction was converted into cDNA using the iScript™ cDNA synthesis kit (Bio-Rad). qRT-PCR was performed using SsoFast™ EvaGreen supermix (Bio-Rad) in a CFX96 Optical Cycler and analysed using the CFX96 manager software (Bio-Rad). Primers were manufactured by Sigma (Table 1) and reactions were optimised and processed according to the manufacturer with initial denaturation/DNA polymerase activation at 95 °C for 30 s followed by PCR: 95 °C for 5 s, variable annealing temperature (see Table 1) for 5 s, and 65 °C for 5 s repeated for 40 cycles. β-actin was used as a housekeeping gene. Data were calculated as relative expressions according to the ΔΔC(t) principle. MiRNAs identified as playing a role in peripheral nerve regeneration were identified by literature review and those selected for assessment included miR-21, miR-222, miR-1, miR-18a, miR-182 [25–29]. The exosomal miRNAs were analysed with Applied Biosystems™ TaqMan™ MicroRNA Assays according the manufacturer’s instructions. No stable housekeeping gene was identified for comparing exosomal miRNAs thus relative levels were expressed in relation to primary Schwann cells (set to value 1). Experiments were performed on 2–3 independent rat preparations with uADSCs and dADSCs samples run in parallel from matched animals.

**Table 1:** Primer sequences for qRT-PCR and annealing temperatures used (°C)

Factor	Forward Primer (5′ → 3′)	Reverse Primer (5′ → 3′)	°C
*Gap43*	GTCCACTTTCCTCTCTATTTC	TGTTCATTCCATCACATTGA	55.0
*Tau*	GGCTACACACCAACCTTTG	CCACCTCCTCCTCACTTC	57.2
*Rac1*	TCATCAGTTACACGACCAATG	ACGCAGTCTGTCATAATCTTC	56.8
*RhoA*	CACACAAGGCGGGAGTTAG	CGTCTTTGGTCTTTGCTGAAC	60.5
*β-actin*	ACTATC GGCAATGAGCGGTTC	AGAGCCACCAATCCACACAGA	65.0

### Exosomal RNA uptake by neurons

Exosomal RNA was fluorescently labelled using SYTO® RNASelect™ green fluorescent cell stain in combination with exosome spin columns according to the manufacturer’s protocol (Invitrogen). The tagged exosomes, and a control of DMEM only, were applied to NG108–15 cells in culture for 3 h before fixation, permeabilisation with 0.1% (*v*/v) Triton X-100 and being mounted with ProLong® gold antifade reagent with DAPI. The chamber slides were viewed with a fluorescence microscope. RNA was isolated (using an miRNeasy kit, Qiagen) from untreated and exosome-treated NG108–15 cells and qRT-PCR performed as described above.

### Statistical Analysis

To determine the statistical difference between the mean ± S.E.M of data sets, one-way analysis of variance (ANOVA) complemented by Bonferroni post hoc test (GraphPad Prism, GraphPad Software) was used. Statistical significance was set as *p < 0.05, **p < 0.01, ***p < 0.001.

## Results

### Stem cell characterisation

**Fig. 1:**
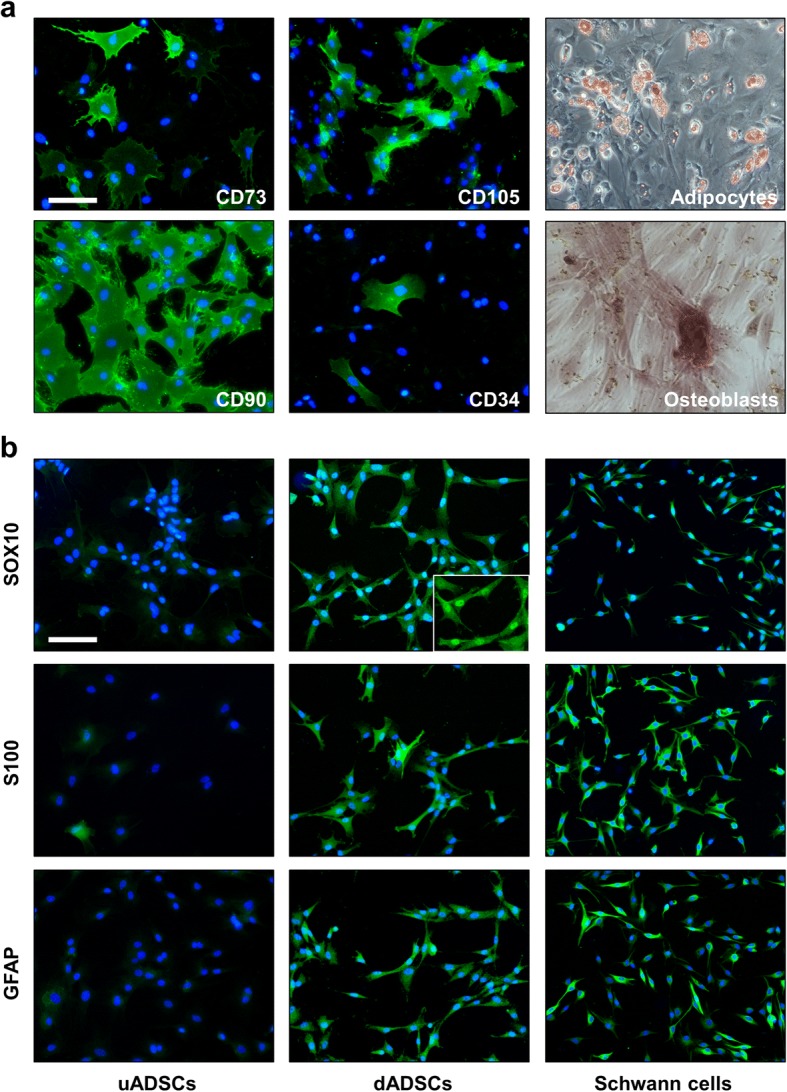
Characterisation of the stem cells. **a** Immunostaining for mesenchymal stem cell markers CD73, CD90, CD105 (green) and putative adipose stem cell potency marker CD34 (green) with DAPI nuclei staining (blue) showing all cells in the field. Cultures could be differentiated into adipocytes (Oil Red O staining for lipids) and osteoblasts (Alazarin Red staining highlighting areas of mineralisation). **b** Undifferentiated adipose stem cells (uADSCs), Schwann-cell like differentiated adipose stem cells (dADSCs) and primary Schwann cells stained with the Schwann cell markers SOX10, S100 and GFAP (green) and DAPI (blue). Insert Sox10 staining shows strong nuclear staining for the transcription factor. Note the elongated morphology of dADSCs, more characteristic of Schwann cells compared with cell shape of uADSCs shown in A. Scale bar in A and B is 50 μm

Cells isolated from rat adipose tissue adhered to tissue culture plastic and expressed the characteristic primary stable positive markers CD73, CD90 and CD105 (Fig. 1a). In addition, approximately 5% of the cells expressed the primary unstable positive marker CD34 at passage 2 (Fig. 1a), one of the markers which distinguishes adipose derived stem cells from those isolated from bone marrow [30]. Exposure of the cultures to adipogenic or osteogenic differentiating media resulted in respective formation of Oil Red O positive lipid droplets and Alazarin Red stained mineral deposits indicating multi-lineage differentiation potential of the cultures (Fig. 1a). The undifferentiated ADSC (uADSCs) cultures were negative for the Schwann cell markers SOX10 and GFAP and approximately 5% were weakly stained with S100B antibody (Fig. 1b). Treatment of the uADSCs with a growth factor stimulation protocol induced the expression of Sox10, GFAP and S100 proteins (Fig. 1b) and morphological changes such that the cells assumed some characteristics of primary Schwann cells (Fig. 1b).

### Exosomes and neurite outgrowth

**Fig. 2:**
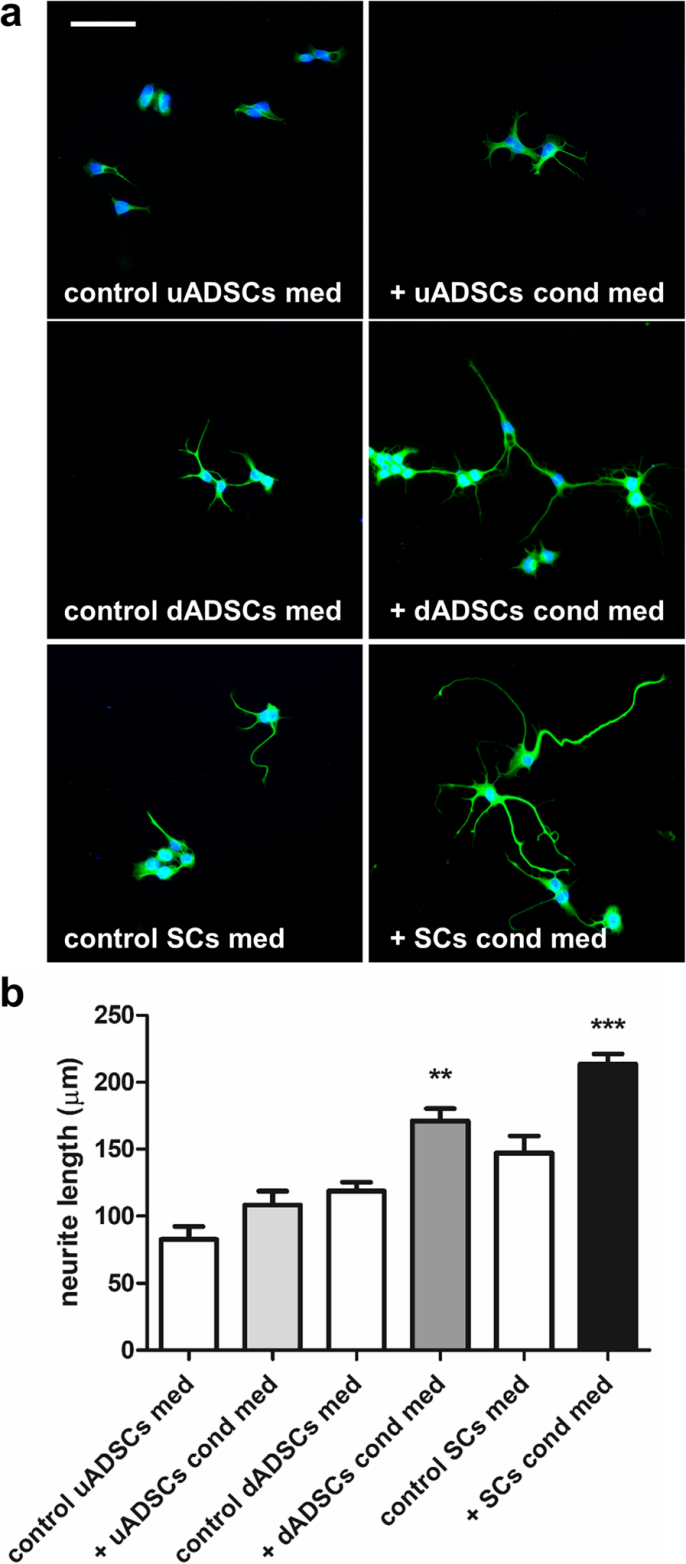
Conditioned media enhances neurite outgrowth. **a** NG108–15 neurons treated with conditioned media from undifferentiated ADSCs (+ uADSCs cond med), Schwann cell-like differentiated stem cells (+ dADSCs cond med) or Schwann cells (+ SCs cond med) stained with βIII-tubulin antibody (green). Control cultures were treated with the respective media for each condition. Scale bar is 100 μm. **b** Neurite length quantification, mean ± SEM, **P < 0.01 and ***P < 0.001 significantly longer neurites compared with respective control media

**Fig. 3:**
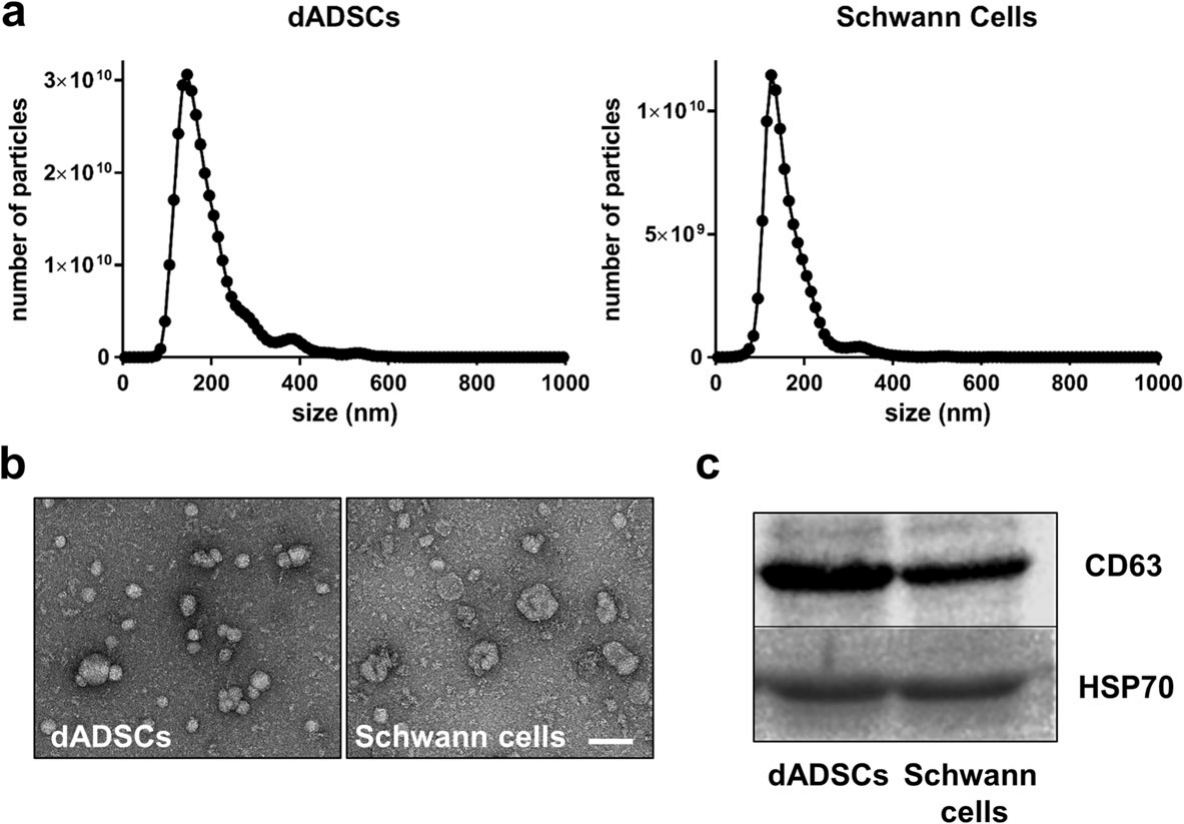
Characterisation of isolated extracellular vesicles. **a** Representative traces from nanoparticle tracking analyses for Schwann cell-like differentiated adipose stem cells (dADSCs) and Schwann cells. **b** TEM analysis of exosome preparations. Scale bar = 100 nm. **c** Western blots showing expression of characteristic exosome markers CD63 and heat shock protein 70 (HSP70) in the extracellular vesicle preparations

**Fig. 4:**
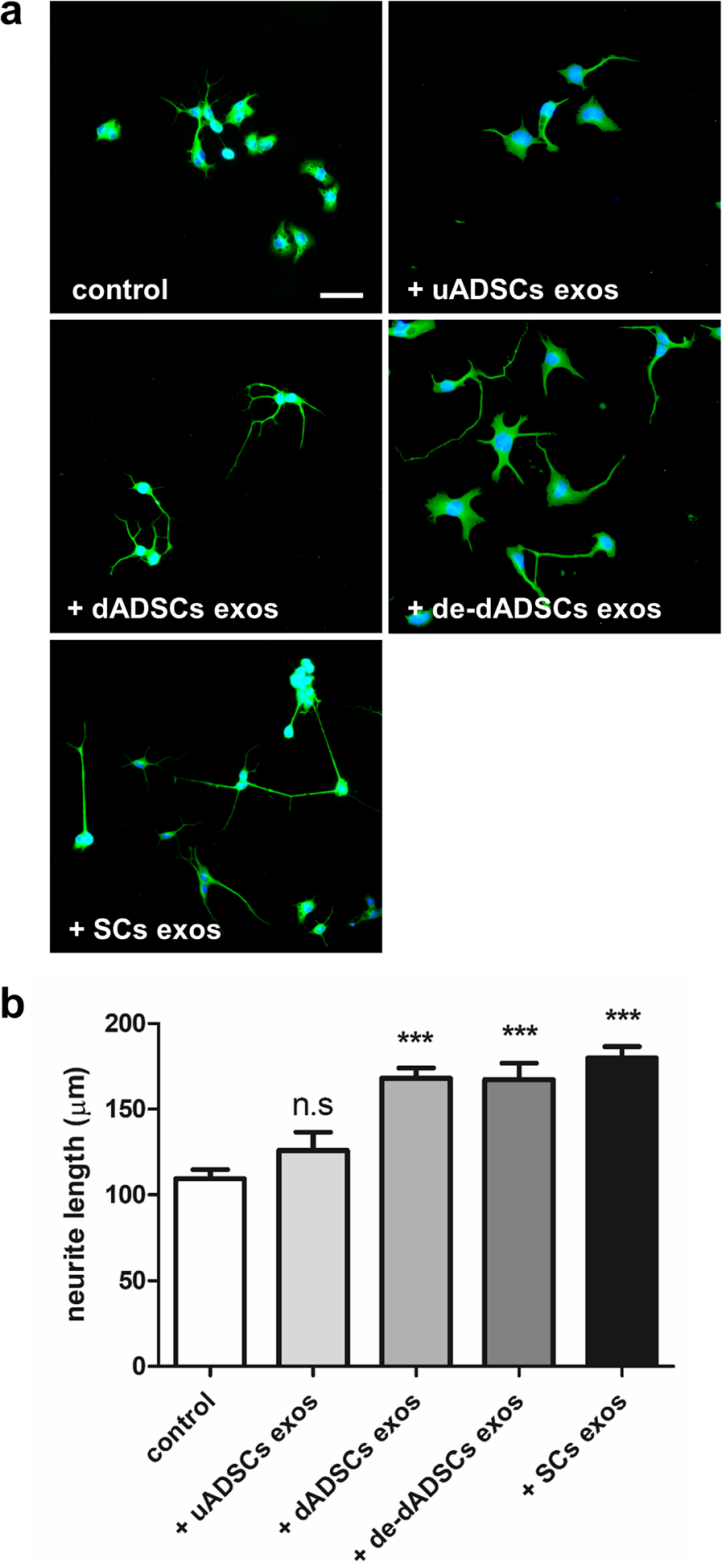
Exosomes enhance neurite outgrowth. **a** NG108–15 neurons treated with exosomes isolated from undifferentiated ADSCs (+ uADSCs exos), Schwann cell-like differentiated stem cells (+ dADSCs exos), dedifferentiated dADSCs (+ de-dADSCs exos) or Schwann cells (+SCs exos) stained with βIII-tubulin antibody (green). Control NG108–15 neurons treated with DMEM only. Scale bar is 100 μm. **b** Quantification of neurite length mediated by dADSCs exosomes, de-dADSCs and Schwann cells (+SCs exos), mean ± SEM, ***P < 0.001 significantly longer neurites compared with control cultures. n.s is not significantly different

The conditioned medium from the Schwann cell-like cultures (dADSCs) significantly increased neurite outgrowth of NG108–15 neurons (171 ± 9 μm v’s 119 ± 7 μm in the respective control cultures; *P* < 0.01; Fig. 2). Primary Schwann cell conditioned media also evoked significant neurite outgrowth (*P* < 0.001; Fig. 2). In contrast, uADSCs media resulted in a non-significant increase in neurite outgrowth (108 ± 10 μm v’s 82.7 ± 9 μm in the respective control cultures; Fig. 2). Following standardised methods for exosome isolation using precipitation, we isolated extracellular vesicles from dADSCs and SCs. Nanoparticle tracking analysis was used to characterise the vesicles produced by both cell types and showed a modal peak size at 132.9 ± 3.59 nm and 124.3 ± 4.10 nm for dADSCs and SCs respectively indicating that these vesicles fall within the size range indicative of exosomes (Fig. 3a). Negative staining and TEM analysis confirmed morphologically the presence of vesicles resembling exosomes in the preparations (Fig. 3b). Furthermore, Western blotting showed that both preparations carried the characteristic exosome markers CD63 and HSP70 (Fig. 3c). Exosomes isolated from conditioned media were applied to NG108–15 cells in culture for 24 h, and compared against control conditions. Exosomes from both dADSCs and SCs significantly enhanced neurite outgrowth (*P* < 0.001) compared with control (Fig. 4). The dADSCs exosomes retained their activity even when the cell cultures were deprived of the stimulating factors (de-dADSCs; 167 ± 10 μm v’s 109 ± 5 μm; P < 0.001). The exosomes from uADSCs produced a small non-significant increase in neurite outgrowth (Fig. 4b).

### Exosome RNA cargo and transfer to neurons

**Fig. 5:**
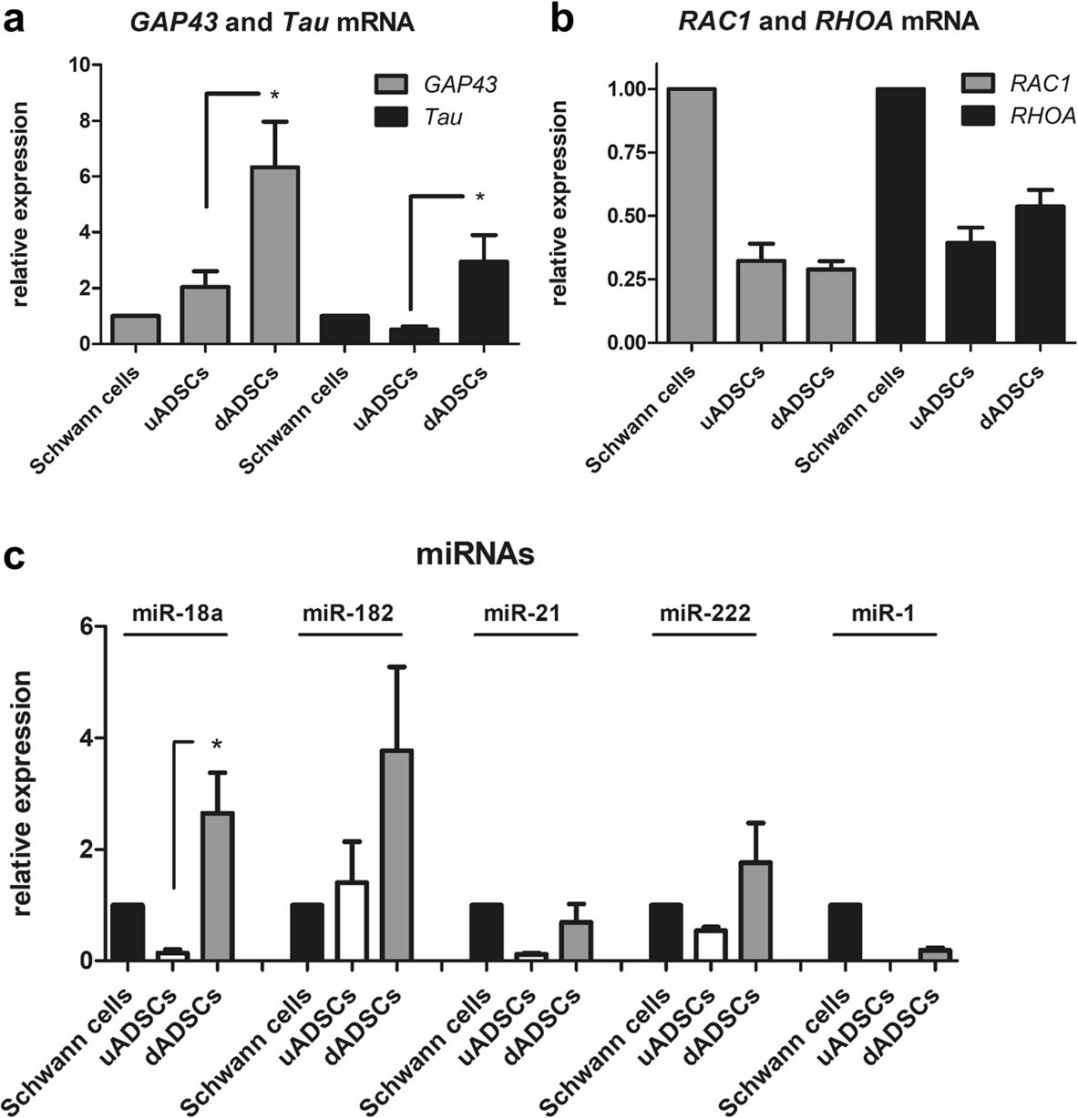
Exosomes express mRNAs and miRNAs associated with neural regeneration. **a** and **b** qRT-PCR was used to measure *Gap43, Tau, Rac1, RhoA* levels in exosome preparations from Schwann cells, undifferentiated adipose stem cells (uADSCs) and Schwann cell-like differentiated adipose stem cells (dADSCs). Expression levels normalised to Schwann cell = 1. *P < 0.05 significant difference in expression levels between the groups shown by connecting lines. **c** qRT-PCR was used to measure miR-18a, miR-182, miR-21, miR-222, miR-1 levels in exosome preparations from Schwann cells, undifferentiated adipose stem cells (uADSCs) and Schwann cell-like differentiated adipose stem cells (dADSCs). Expression levels normalised to Schwann cell = 1. *P < 0.05 significant difference in expression levels between the groups shown by connecting lines

Significant quantities of total RNA were extracted from SCs and both uADSCs and dADSCs derived exosomes. Based on literature reviews we proceeded to identify exosomal mRNAs and miRNAs that are involved in axonal growth and regeneration. Using qRT-PCR, it was shown that both *Gap43* and *Tau* mRNA were significantly (*P* < 0.05) upregulated in dADSCs versus uADSCs and showed approximately 6 and 3-fold higher expression in dADSCs compared with primary Schwann cells (Fig. 5). *Rac1* and *RhoA* were detected in the stem cell derived exosomes to a lower extent than found in the Schwann cell exosomes, although this was not found to be significant (Fig. 5). MiRNAs previously shown to have enriched expression in axons (miR18a and miR-182) and to be promoters of nerve regeneration and neurite outgrowth (miR-21 and miR-222) were detected in dADSCs and primary Schwann cell-derived exosomes (Fig. 5). All four miRNAs were up-regulated by the differentiation process showing higher levels of expression than uADSCs (Fig. 5). MiR-1, another miRNA shown to be dynamically regulated upon peripheral nerve injury was undetectable in uADSCs and showed considerably lower expression levels in dADSCs compared with SCs (Fig. 5).

**Fig. 6:**
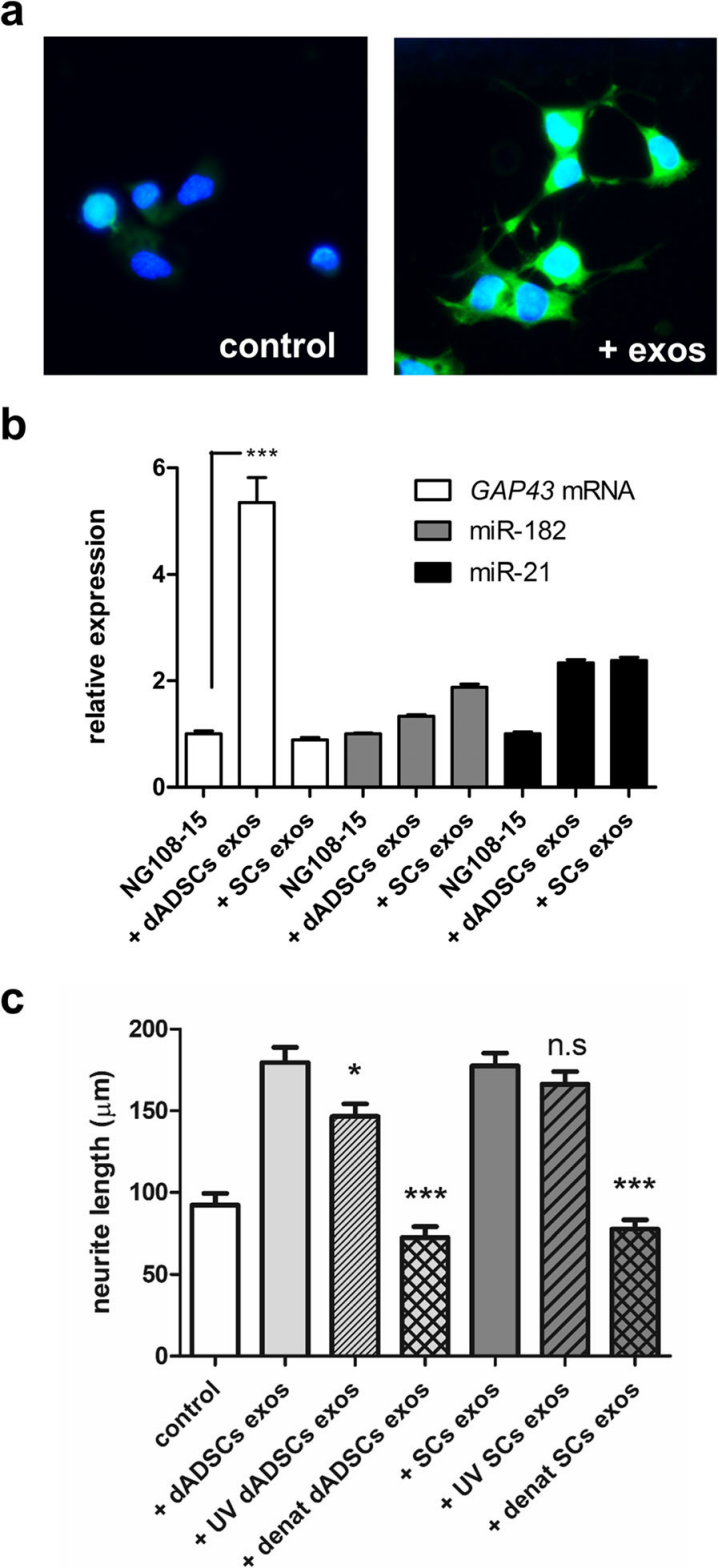
Exosomes transfer RNAs to neurons and this is partly responsible for mediating neurite outgrowth. **a** Exosomes were labelled with SYTO® RNASelect™ green fluorescent dye and applied to NG108–15 neurons (+ exos). Control cultures were treated with DMEM. DAPI blue staining shows cell nuclei. **b** qRT-PCR was used to measure *Gap43* mRNA, miR-182, and miR-21 levels in control NG108–15 cultures and those treated with Schwann cell-like differentiated adipose stem cell derived exosomes (+ dADSCs exos) or Schwann cell derived exosomes (+SCs exos). Relative expression levels compared with control NG108–15 cultures = 1. ***p < 0.001 significantly elevated levels compared with the control cultures. **c** Quantification of neurite outgrowth mediated by exosomes derived from Schwann cell-like differentiated adipose stem cells (+ dADSCs exos) and dADSCs derived exosomes treated with UV irradiation (+ UV dADSC exos). Parallel experiments were performed with exosomes from Schwann cells (+ SCs exos) and UV irradiated Schwann cell-derived exosomes (+ UV SCs exos), mean ± SEM, *p < 0.05 significantly shorter neurites in the presence of UV treated dADSCs exosomes compared with untreated dADSCs exosomes**.** n.s is not significantly different. Exosomes preparations were also heated to 98 °C for 10 min to denature the exosomal proteins (+ denat dADSCs exos, + denat SCs exo; ***p < 0.001 significantly shorter neurites compared with untreated exosomes)

The transfer of exosomal RNAs to neurons was confirmed by fluorescently tagging the RNA of exosomes in suspension and then applying to NG108–15 cells. Analysis by microscopy showed that the internalised exosomal RNA was distributed throughout the nerve axons, and also at the cell body (Fig. 6a). NG108–15 cultures treated with dADSCs derived exosomes showed significantly (*P* < 0.001) greater than 5-fold higher expression levels of *Gap43* mRNA and also miR-182 and miR-21 levels were elevated in NG108–15 cells treated with the exosomes further suggesting the transfer of RNA molecules (Fig. 6b). Given these observations we hypothesised that the effects of exosomes on neurite outgrowth could be mediated by RNA transfer. To test this hypothesis, exosomes were exposed to UV-light for 2 × 30 min, as UV-light inactivates RNA functions. Compared with control dADSCs-derived exosomes the UV treated dADSCs exosomes showed significantly reduced (*P* < 0.05) effects on neurite outgrowth (Fig. 6c). However, there was no effect of UV-irradiation on SCs-derived exosomes. Denaturation of exosomal proteins completely eliminated the increases in neurite outgrowth (Fig. 6c).

## Discussion

Following peripheral nerve injury, Schwann cells coordinate the regeneration of axons. Their secretome, which includes traditionally described paracrine neurotrophic substances (e.g. nerve growth factor; NGF and brain derived neurotrophic factor; BDNF), is largely responsible for this [31, 32] but the means by which this is coordinated is still debated. Application of SCs as part of surgical nerve repair enhances axon regeneration in experimental *in vivo* models [33] but to date this procedure has not reached large scale clinical evaluation as it does not match or exceed the results of autologous nerve grafting and still does not overcome the need for sacrifice of healthy nerve tissue. Thus, an alternative method, still at the pre-clinical stage, is to use stem cells which have been differentiated to mimic the properties of SCs. In this study we used a differentiation protocol which we first described for ADSCs in 2007 [19], based on similar findings in bone marrow stromal/stem cells [20]. Since then, there have been a number of studies which have investigated further the role of the individual components and the timing of their application [34, 35]. In addition to expressing glial cell markers the stimulated ADSCs have also been shown to express specific peripheral myelin proteins and can myelinate dorsal root ganglia neurons [36, 37]. The treated ADSCs promote nerve regeneration *in vivo* [4, 6, 38] and this is likely, in large part, due to their rich secretome of neurotrophic and angiogenic factors [39]. We showed that conditioned medium from the dADSCs significantly enhanced neurite outgrowth whereas undifferentiated stem cells had little effect and this confirms our own, and other research groups, previous reports [19, 40]. The effects of dADSCs have been shown to be, in part, mediated by classic secreted paracrine factors such as BDNF and NGF [40]. Recent understanding of the role of secreted exosomes in cell-to-cell communication, as an alternative to the traditional paracrine signalling processes, has led to the concept of them as potential therapeutic agents to treat various clinical conditions including nerve injury [41]. Previous studies have shown that exosomes are produced by SCs, internalised by injured neurons and can enhance axon regeneration [18]. Further investigation of their properties will likely allow adaptation and refinement to boost their potential to treat nerve injuries.

In this study we showed that adipose stem cells that have been differentiated into a Schwann cell-like phenotype (dADSCs) secrete exosomes, like their primary SCs counterparts, and these enhance *in vitro* neurite outgrowth. Importantly, the dADSCs continue to produce exosomes which have high neurite outgrowth promoting activity, even in the absence of the stimulating factors. This differs from a study by Faroni et al. [42] which showed that withdrawal of the factors led to rapid downregulation of secreted paracrine neurotrophic factors. The importance of the stimulating/differentiating protocol is highlighted by the fact that exosomes from uADSCs did not evoke significant increases in neurite outgrowth. This is confirmed by a recent study showing that undifferentiated ADSCs exosomes have a very limited effect on DRG neurite outgrowth, in contrast to conditioned media treatment [43].

In order to further investigate the role of exosomes in nerve injury and identify how they could be used therapeutically, it is imperative to understand the cargo they carry and what effect it could have on recipient cell function. Extracellular vesicles of all cell types tested have been shown to carry proteins [44] and RNAs [45] to targeted recipient cells. When these are internalised they can affect that cell function, altering its phenotype [12]. The RNAs transferred are of various types; mRNAs and miRNAs amongst them. The mRNAs have the ability to translate proteins, and the miRNAs the ability to suppress protein production by binding with endogenous cell mRNA and causing its degradation or post-transcriptional suppression. MicroRNAs are short (21–22 nucleotides) non-coding RNAs that bind with corresponding segments on mRNAs [46, 47], and miRNAs found in both dorsal root ganglia neurons and SCs have been shown to vary in expression following nerve injury [25, 48]. Evidence supports a role for miRNAs in the dedifferentiation of Schwann cells to a non-myelinating phenotype during Wallerian degeneration and as such as modulators of the Schwann cell response to neuronal injury [49]. Additionally, miRNAs affecting cytoskeletal organisation are found in abundance in the axon or nerve terminal [50] indicating a local control over axonal growth.

Our results showed that the exosomes isolated from SCs and dADSCs, contained messenger and microRNAs that are known to play roles in nerve regeneration. Of these, GAP43 is a neural growth-associated protein which is important in translating signals required for growth cone guidance, with overexpression leading to increased neurite sprouting [51]. Tau protein interacts with tubulin to maintain the stability of the microtubule structure. Tau expression decreases day 1 post-injury but then steadily increases to a maximum concentration at day 14; these findings indicate a strong relationship with the regeneration process. The fact that the exosomes from dADSCs showed upregulated *Tau* and *Gap43*-coding mRNAs could be an important element resulting in the increased outgrowth seen in the experiments. RAC1, a member of the Rho GTPase family, is a protein that has a role in the control of actin dynamics. It is essential for cell proliferation and migration, and is subsequently required for nearly all aspects of neuronal regeneration. Deletion of the gene coding for this protein leads to neuronal loss and accelerated cell cycle exit [52]. Results from this study showed high levels of the mRNA for RAC1 in SCs exosomes, which suggested a likely role of these vesicles in the regeneration process. Presence of this mRNA was shown in exosomes from both uADSCs and dADSCs but at much lower levels compared with SCs. RhoA, like RAC1, is a small GTPase but unlike RAC1 is a suppressor of axon regeneration. It has been shown to limit recovery by evoking neuronal apoptosis and regenerative failure through growth cone collapse [53–55]. It was therefore surprising to find the mRNA coding for this protein was highly expressed in SCs exosomes.

The miRNAs miR-18a and miR-182 were shown to be present in exosomes derived from SCs, uADSCs and dADSCs. These miRNAs are enriched in axons [29] and their presence in the exosomes suggests that they could play a role in axon regeneration via direct transfer at the growth cones. Specific targets of these small RNAs are not yet clear. miR-222 promotes Schwann cell proliferation and migration by targeting longevity assurance homologue 2 (LASS2) which suppresses cell growth [56], promotes neurite outgrowth with increased expression directly targeting phosphatase and tensin homolog (PTEN), a known inhibitor of nerve regeneration [26] and, in addition to miR-21, inhibits apoptosis of neurons following injury by suppressing tissue inhibitor of metalloproteinase 3 (TIMP3), a pro-apoptotic protein [57]. miR-21 also downregulates a further inhibitor of nerve regeneration, Sprouty2 [58]. As miR-222 and miR-21 were shown to be present in SC exosomes, the mechanism through which SCs support the regeneration of injured neurons could involve the exosomal transfer of these miRNAs. Furthermore, both uADSCs and dADSCs exosomes contained these miRNAs, with an increased expression noted upon differentiation. The presence of these miRNAs in exosomes from dADSCs indicates that these vesicles could mimic the SCs role in aiding regeneration by down-regulating intrinsic inhibitors of regeneration. In addition to the aforementioned potential positive regulators of axon regeneration we identified miR-1 expression in SCs exosomes and to a significantly lesser extent in the dADSCs derived exosomes. BDNF, an important modulator of Schwann cell-mediated axon regeneration, is a target of miR-1 [27] and the silencing of miR-1 increases SCs proliferation. Thus, to fully utilise exosomes for nerve regeneration it might be necessary to load them with selected miR-1 antagomirs to block their possible anti-regenerative functions.

Importantly our experiments strongly suggested that it was the RNA molecules contained with the dADSCs exosomes that played a role in the effects on neurite outgrowth. UV-irradiation which damages genetic material, reduced the potency of the exosomes derived from dADSCs. So how might the transferred RNA molecules affect neurite outgrowth? In 2010, Yoo et al. [59] showed evidence supporting both temporal as well as spatial control over protein synthesis in peripheral nerve regeneration. Messenger RNAs were shown to be stored in dormant forms in the distal axon until they were stimulated when needed for regeneration. Local translation was activated upon nerve injury with increased NGF and BDNF leading to additional axonal transport of β-actin mRNA. These observations support the idea that genetic control of the regenerating growth cone is a local process. Our results with the dADSCs exosomes suggest that the transfer of external RNAs could modulate these effects. However, it appears that SCs exosomes modulate neurite outgrowth via RNA independent mechanisms and denaturing the exosomal proteins completely eliminated the neurite outgrowth promoting effects of SC-derived exosomes. Interestingly, the same procedure also fully attenuated the effect of dADSCs exosomes suggesting that this method also interfered with the RNA mechanism which is in contrast to a study which showed that only combined RNA and protein inhibition worked to significantly eliminate functional effects of exosomes [60].

The therapeutic potential of using dADSCs derived exosomes as surrogates for SCs in supporting nerve regeneration is well-supported by the findings of this study. One careful consideration that needs to be taken is the fact that exosomes are representatives of their parent cell, in that the state of that cell is reflected in the cargo of the vesicles. As such, it is not reliable that every dADSCs exosome would contain the same exact contents as others, which could cause unexpected and unfavourable results. Furthermore, given that the starting cell populations are highly heterogeneous, we cannot rule out the possibility that some of the cells in the mix which do not differentiate into the SC-like phenotype contribute to the outcomes described in this study. The starting stromal vascular fraction extracted from adipose tissue consists of many different cell types in addition to the ADSCs including endothelial progenitor cells, smooth muscle cells, immune cells and fibroblasts but the heterogeneity of the cultures is progressively reduced by washing and culture in stem cell supportive media [61, 62]. We begin the ADSCs-to-Schwann cell differentiation process at passage 2, at which stage we are unable to detect surface markers representative of immune or endothelial cells (data not shown). After 2 weeks stimulation, the differentiation protocol results in a majority of the cells expressing glial cell markers. Therefore we feel confident that the exosomes that we collect from these differentiated cultures originate from the dADSCs and the corresponding described RNA cargoes are also representative of the dADSCs, not any contaminating non-ADSCs.

Although these experiments have identified that their cargo does include factors important in peripheral nerve regeneration, the exosomes might need to be further tailored with exogenously loaded miRNAs and antagomirs to reach their full potential. Furthermore, development of new protocols for methods such next generation RNA sequencing technology will allow detection of all RNA subtypes in the exosome as well as unannotated transcripts and allow identification of other low-abundance RNAs.

The potential benefits of exosomes versus “live stem cell therapy” is that they do not need to be autologously derived due to their immunologically inert features. They could be harvested in the laboratory from discarded adipose tissue and stored ready to be used later. In the next step our future translational studies will investigate the *in vivo* effects of the exosomes in different types of nerve injury model. We will need to address various clinically relevant parameters such as dosing, timing and method of exosome delivery. Lopez-Verrilli et al. injected crushed rat sciatic nerves with SC exosomes directly into the distal stump at daily intervals for four days, and showed an increase in outgrowth when compared with controls [18]. Translation of this to humans could be through the use of ultrasound-guided injections, although this would be painful and labour-intensive. The choice of exosomal cargo and application site could be altered according to time, providing a cocktail of elements needed specific to the stage of regeneration. For those patients with nerve gaps, these exosomes could be partnered with the use of artificial conduits in a one-stage procedure that guides axonal growth through close contact in the tube.

## Conclusions

In summary this work shows how dADSCs exosomes can mimic the positive effects of SCs exosomes on neurite outgrowth and transfer of RNA molecules from dADSCs derived exosomes plays an important role in the process. Exosomes represent an important component of the stem cells secretome which have great potential for further manipulation tailored for the treatment of nerve injuries.

## Abbreviations

BDNF: brain derived neurotrophic factor
dADSCs: Schwann cell-like differentiated adipose derived stem cells
DMEM: Dulbecco’s Modified Eagle’s Medium
FCS: foetal calf serum
GFAP: glial fibrillary acidic protein
MEM: Minimum Essential Medium
SCs: Schwann cells
uADSCs: undifferentiated stem cells
